# Adapting a Mobile Health App for Smoking Cessation in Black Adults With Anxiety Through an Analysis of the Mobile Anxiety Sensitivity Program Proof-of-Concept Trial: Qualitative Study

**DOI:** 10.2196/53566

**Published:** 2025-02-07

**Authors:** Marshall K Cheney, Adam C Alexander, Lorra Garey, Matthew W Gallagher, Emily T Hébert, Anka A Vujanovic, Krista M Kezbers, Cameron T Matoska, Michael J Zvolensky, Michael S Businelle

**Affiliations:** 1 Department of Health and Exercise Science University of Oklahoma Norman, OK United States; 2 Tobacco Settlement Endowment Trust (TSET) Health Promotion Research Center University of Oklahoma Health Sciences Center Oklahoma City, OK United States; 3 Department of Psychology University of Houston Houston, TX United States; 4 Helping Everyone Achieve a LifeTime of Health (HEALTH) Institute University of Houston Houston, TX United States; 5 Department of Health Promotion and Behavioral Sciences UTHealth School of Public Health Austin, TX United States; 6 Department of Psychological and Brain Sciences Texas A&M University College Station, TX United States; 7 Department of Behavioral Science The University of Texas MD Anderson Cancer Center Houston, TX United States; 8 Department of Family and Preventive Medicine University of Oklahoma Health Sciences Oklahoma City, OK United States

**Keywords:** cultural tailoring, tailoring, African American, black, smoking cessation, mHealth, smartphone application, just in time adaptive intervention, qualitative, formative evaluation, app, application, anxiety, adult, qualitative analysis, smoking, mobile phone, tobacco

## Abstract

**Background:**

At least half of smokers make a serious quit attempt each year, but Black adults who smoke are less likely than White adults who smoke to quit smoking successfully. Black adults who smoke and have high anxiety sensitivity (an individual difference factor implicated in smoking relapse and culturally relevant to Black adults) are even less successful. The Mobile Anxiety Sensitivity Program for Smoking (MASP) is a smoking cessation smartphone app culturally tailored to Black adults who smoke to increase smoking cessation rates by targeting anxiety sensitivity.

**Objective:**

This study examined the acceptability and feasibility of the MASP smartphone app following a 6-week pilot test through postintervention qualitative interviews.

**Methods:**

The MASP smoking cessation app was adapted from an evidence-based app by adding culturally tailored narration and images specific to the Black community, educational content on tobacco use in the Black community and the role of menthol, culturally tailored messages, and addressing tobacco use and racial discrimination. The MASP app was piloted with 24 adults with high anxiety sensitivity who identified as Black, smoked daily, and were not currently using medications or psychotherapy for smoking cessation. At the end of the 6-week pilot test, 21/24 participants (67% female; 95.2% non-Hispanic; mean age=47.3 years; 43% college educated; 86% single or separated) completed an audio-recorded semistructured interview assessing the acceptability and utility of the app, individual experiences, barriers to use, the cultural fit for Black adults who wanted to quit smoking, and identified areas for improvement. Transcribed interviews were coded using NVivo (Lumivero), and then analyzed for themes using an inductive, use-focused process.

**Results:**

Most participants (17/21, 81%) had smoked for more than 20 years and 29% (6/21) of them smoked more than 20 cigarettes daily. Participants felt the MASP app was helpful in quitting smoking (20/21, 95%) and made them more aware of smoking thoughts, feelings, and behaviors (16/19, 84%). Half of the participants (11/21, 52%) thought the combination of medication and smartphone app gave them the best chance of quitting smoking. Themes related to participant experiences using the app included establishing trust and credibility through the recruitment experience, providing personally tailored content linked to evidence-based stress reduction techniques, and self-reflection through daily surveys. The culturally tailored material increased app relevance, engagement, and acceptability. Suggested improvements included opportunities to engage with other participants, more control over app functions, and additional self-monitoring functions.

**Conclusions:**

Adding culturally tailored material to an evidence-based mobile health (mHealth) intervention could increase the use of smoking cessation interventions among Black adults who want to quit smoking. Qualitative interviews provide mHealth app developers important insights into how apps can be improved before full study implementation and emphasize the importance of getting feedback from the target population throughout the development process of mHealth interventions.

**Trial Registration:**

ClinicalTrials.gov NCT04838236; https://clinicaltrials.gov/ct2/show/NCT04838236

## Introduction

Black or African American (hereafter referred to as Black) adults who smoke experience significant tobacco-related health disparities [1]. Black adults who smoke are at higher risk for tobacco-related death and disease, including lung cancer [2,3], heart disease, and stroke [4]. On average, Black adults who smoke initiate smoking later in life and smoke fewer cigarettes per day relative to their European American or White counterparts [5,6]. Yet, Black adults who smoke demonstrate greater cigarette dependence and are less likely to quit relative to other racial and ethnic groups [7,8], despite engaging in more quit attempts [8,9]. The difficulty this group faces in quitting successfully highlights the wide range of tobacco-related disparities that disproportionately impact Black adults who smoke (eg, tobacco advertising in predominately Black neighborhoods) [10-13].

One additional factor that can influence the success of a quit attempt and sustained smoking cessation among Black adults who smoke is anxiety sensitivity. Anxiety sensitivity is a relatively stable individual trait predisposing individuals to focus on internal bodily feelings of distress and to believe that anxiety and the bodily symptoms they experience from anxiety are uncommon or indicate an underlying threat to their health [14,15]. People who smoke and have high anxiety sensitivity can experience higher nicotine withdrawal symptoms and more intense cravings when they quit smoking [16], they are more prone to smoke when they have smoking-related thoughts, feelings, and sensations during cessation attempts, and they are more likely to smoke again soon after they have initiated a quit attempt [17-19]. Anxiety sensitivity is prevalent in up to a third of people who smoke across all races and ethnic groups [20,21]; however, Black people who smoke and have higher levels of anxiety sensitivity report experiencing the internal physical symptoms of nicotine withdrawal more intensely than Hispanic and White people who smoke [22,23]. Black people who smoke were also more likely to perceive these symptoms were out of their control or were worsened by environmental stressors such as racial discrimination [22,23]. Such processes could contribute to Black people being less successful in quitting smoking or put them more at risk of returning to smoking to help alleviate stress and anxiety [19].

One promising smoking cessation strategy for Black people with anxiety sensitivity is the delivery of tailored interventions through smartphone apps. Mobile health (mHealth) interventions can provide low-cost access to evidence-based smoking cessation strategies that extend the reach of traditional smoking cessation interventions. mHealth interventions can increase access for people who smoke but do not wish to engage in traditional smoking cessation interventions, those not interested in face-to-face interventions, and those who live in remote or underserved areas [24,25]. Smartphone smoking cessation apps have high acceptability in people who wish to quit smoking [25-27] and some smoking cessation apps have demonstrated efficacy in helping people to quit smoking, particularly for the most engaged app users [24,28-30]. While Black adults are underrepresented in mHealth studies [31], Black adults who have participated in mHealth studies viewed the interventions positively and found them to be acceptable and easy to use [32]. Thus, mHealth approaches may also reduce disparities in smoking cessation rates for Black people who smoke [33].

A benefit of mHealth smartphone apps is their potential to tailor intervention content to increase its relevance and acceptability for people who smoke. Tailoring involves selecting intervention content that corresponds to participant characteristics such as beliefs, identity, or readiness to change that can influence the success of a behavior change intervention and provide relevant content in the moments it is needed most [25,34-36]. Specifically, tailoring is the process of modifying the specific content a person receives in a mHealth smoking cessation intervention through *personalization* (based on regular participant assessments) and *feedback* (presenting participants with information about their behaviors over the course of the intervention) [34].

Cultural tailoring is an additional tailoring process of modifying an evidence-based mHealth behavioral intervention to increase the cultural relevance or cultural sensitivity of the intervention to a specific cultural group [37,38]. Cultural tailoring can include adjusting or adding intervention content to better match cultural and historical experiences, social norms and values, and meanings of tobacco use to a specific cultural group [37]. Culturally tailored mHealth interventions can also address unique risk and protective factors, incorporate more inclusive language, and include culturally specific images and messages to decrease problems of acceptability, inclusion, and engagement [25,35,38,39]. Although it is not clear if culturally tailored materials increase the ultimate effectiveness of interventions, previous work has shown that Black people who smoke preferred culturally tailored content over standard content, which could ultimately increase acceptability and engagement with smoking cessation interventions, and improve retention rates [39-43]. Pilot testing culturally tailored interventions before full study implementation can verify if adapted content is acceptable and relevant to a cultural subgroup and can be used to obtain feedback on ways to improve the cultural relevance of the intervention [37,44].

The purpose of this study was (1) to assess participant reactions to an evidence-based smoking cessation smartphone app that was culturally tailored for Black people who smoke and have high anxiety sensitivity and (2) to provide recommendations for additional modifications to the app before full study implementation.

## Methods

### Mobile Anxiety Sensitivity Program for Smoking Adaptation

**Figure 1:**
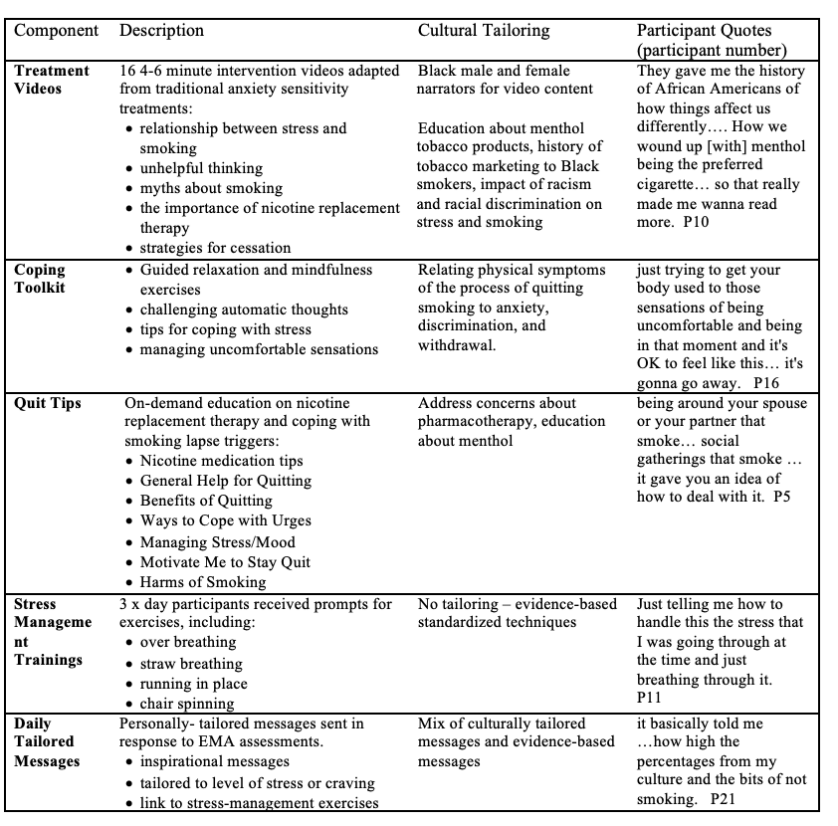
MASP (Mobile Anxiety Sensitivity Program) app components and cultural tailoring.

The Mobile Anxiety Sensitivity Program for Smoking (MASP) is a 6-week smoking cessation smartphone app for Black people with high anxiety sensitivity that was adapted from an evidence-based mHealth smartphone app for people with anxiety sensitivity [30,36,45,46]. The app contained a balance of standard intervention content for all participants (ie, on-demand tips, videos, and guided exercises) and individually tailored content based on the participant’s responses to baseline, daily, and weekly assessments (ie, just-in-time messages; Figure 1) [46].

Based on the model proposed by Barrera and Castro [37], the MASP app was adapted for Black people who smoke with high anxiety sensitivity in an initial pilot study by incorporating culturally tailored content based on theoretical and empirical guidelines [47]. The MASP app was then reviewed by the University of Houston HEALTH Research Institute Community Research Advisory Board, which is part of a broader U54 addressing health equity. The Community Research Advisory Board provided input into video content and narration, clearer and more engaging language, and recruitment materials and strategies. From this input, key MASP app features were revised to include more culturally relevant content including treatment videos, messages, and on-demand content (Figure 1 and Multimedia Appendices 1 and 2). Examples of culturally tailored content included using Black male and female voices to narrate the videos, drawings, and illustrations of Black artists, the inclusion of additional videos addressing menthol tobacco products, the history of tobacco marketing to Black adults, and the impact of racism and discrimination on stress, smoking, and smoking relapse (Figure 1).

Expanded message content was created that related physical symptoms of quitting smoking to anxiety, discrimination, and severity of withdrawal symptoms, as well as messages relevant to Black culture (Textbox 1). Expanded educational content addressed topics relevant to Black people who smoke, including concerns about nicotine replacement therapy and medications to quit smoking. Several areas of the app were not modified, including skills-based approaches such as standard interceptive stress exposure videos, because of the universal applicability of the content [46]. The revised app reflected a balance of culturally tailored and standard material, consistent with other culturally adapted interventions [43].

**Examples of Mobile Anxiety Sensitivity Program culturally tailored messages for Black smokers.**

- Your Tip: (FirstName), Did you know that 45,000 Black people die from smoking-related diseases each year in the US?
- Your Tip: (FirstName), 4 out of 5 Black smokers buy menthols. Chemicals are added to menthol cigarettes to give them a fresh, minty taste. This can make it easier for a smoker to inhale deeply, which may allow more harmful chemicals to enter the lungs.
- Your Tip: (FirstName), Did you know that the money that Black smokers spend on cigarettes in a single day could send 2500 Black students to college for an entire year?
- Your Tip: (FirstName), Tobacco companies have pushed menthol products on the Black community for decades. Did you know that there are significantly more menthol advertisements at stores in neighborhoods with a higher proportion of African American residents?
- Your Tip: (FirstName), Each year smoking kills more African Americans than AIDS, car accidents, fires, alcohol, drugs, and guns combined!
- Your Tip: (FirstName), Did you know African American children and adults are more likely to be exposed to secondhand smoke than any other racial or ethnic group? By quitting smoking you’re not only making life better for yourself, but for the people around you too.
- Your Tip: (FirstName), Tobacco companies have targeted the African American community for decades. By quitting smoking, you’re not only standing up for yourself but also for the members of your community.

### Recruitment

Participants were recruited through Facebook (Meta) advertisements, mass emails through the University of Houston, and fliers posted in organizations and businesses in Houston communities with high concentrations of Black people. Individuals who saw the recruitment materials accessed the screening questionnaire through a posted link on the recruitment materials to assess their eligibility for the study. Individuals were eligible to participate in the pilot study if they (1) self-identified as Black or African American, (2) smoked daily (at least 10 cigarettes per day) for at least 2 years, (3) reported high Anxiety Sensitivity (Short Scale Anxiety Sensitivity Index score of at least 5), (4) endorsed a desire to quit smoking 2 weeks after completion of the baseline assessment, (5) were fluent in English, (6) had no current use of pharmacotherapy or psychotherapy for smoking cessation, and (7) had no cognitive impairment.

Fully enrolled participants (N=24) were instructed to download the INSIGHT (Insight Enterprises Inc) app to their personal smartphone, and those without a compatible phone were provided a study smartphone loaded with the MASP app. All participants were then asked to complete the baseline assessment in the app. Following baseline assessments and a final phone call appointment with project staff members, participants initiated the 6-week intervention. During the 6-week intervention, participants received 5 daily brief assessments (ie, 1 in the morning, 1 in the evening, and 3 at random times throughout the day) followed by individually tailored smoking cessation messages. Along with the daily surveys, participants were prompted to complete weekly follow-up assessments and self-reported smoking status. Participants also had access to stress management and relaxation videos, on-demand tips, strategies for smoking cessation and coping with cravings and urges, and no-cost nicotine replacement therapy. At the end of the intervention, participants used the app to complete a final assessment survey and were asked to participate in an online interview about their experiences with the app [46]. At the end of the 6-week intervention, participants were compensated US $45 for completing the week 6 survey, carbon monoxide breath test, and qualitative interview [46].

### Qualitative Interviews

At the end of the 6-week pilot test, 21 of 24 (88%) participants agreed to participate in an interview about their experiences with the app. The interviews took place between March and August 2022. Two interviewers trained in qualitative interviewing techniques conducted the interviews over Zoom (Zoom Video Communications). The interviews with individual participants lasted an average of 44 minutes each. The interview question path was based on a previous study [48] and adapted to address the acceptability and utility of the app for smoking cessation, individual experiences with each component of the app, barriers to use, the relevance and fit for Black people who smoke, and identify areas for revision before full study implementation (Textbox 2 for question path excerpts).

**Excerpts from the Mobile Anxiety Sensitivity Program interview question path.**

- What could have been done differently to help you complete more of the daily surveys?
- What are some specific things you liked about the app?
- What things did you like least about the app?
- If you had a slip-up during your quit attempt, was there ever a time that you didn't record that on the app?
- You received some different messages from the app during the study. Can you tell me about a time when you found the message to be really helpful when coping with stress?
- Can you tell me about a time when you found the message you received to be really helpful for quitting smoking?
- If you could add one thing to the app to make it more helpful to future smokers, what would you add?
- How relevant do you think the app is for Black Americans or African Americans who want to quit smoking?
- Do you have any suggestions for any way that we can improve the cultural tailoring of the app for Black Americans or African American smokers?

The audio-recorded interviews were transcribed verbatim, reviewed against the audio recording for accuracy, and then imported into NVivo (version 11; Lumivero) for data analysis. A qualitative researcher (MKC) first read through the transcripts, developed the codebook, and coded the transcripts in 2 coding passes. The codebook was developed using a usage-focused lens [49]. For the first coding pass, the individual interview questions were used as codes, which captured the specific functions of the MASP app, how participants interacted with the app, response to cultural tailoring, and the relevance of app content to Black people who wanted to quit smoking. The second coding pass used a pragmatism lens, coding for additional meanings and uses of the app across interview questions [50,51]. Following interview coding, a thematic analysis was conducted. The coded material was reviewed first within each code, then in groupings of related codes, and finally across all codes. Data displays were also created by grouping participant responses over a set of interview questions or codes to assess additional patterns of responses for individual participants [52]. A second qualitative researcher (AA) read the transcripts, reviewed the coding and primary data displays, and provided additional feedback. The qualitative researchers then arrived at the final determination of themes and quotes through an iterative process of feedback and discussion. A final review of the transcripts was then conducted for confirming and disconfirming evidence of themes [52]. Representative quotes were independently selected and distributed across participants.

### Ethical Considerations

The University of Houston (reference number: 12747) and the University of Oklahoma Health Sciences Center (reference number: STUDY00000360) institutional review boards approved the protocol presented in this study. This trial was registered at

ClinicalTrials.gov (NCT04838236).

## Results

### Participant Characteristics

Most (15/21, 71%) of the participants who completed the qualitative interviews were age 40 years or older (mean 47.33, SD=9.6), almost half (9/21, 43%) were college graduates, two-thirds were female (14/21, 67%), most participants were non-Hispanic (20/21, 95%), and reported being single (18/21, 86%). The majority smoked less than a pack of cigarettes per day (15/21, 71%), had smoked for 20 years or longer (17/21, 81%), smoked menthol cigarettes (20/21, 95%), and smoked an average of 15.48 (SD 5.02) cigarettes per day. Participants had moderate levels of nicotine dependence (mean 3.10, SD 1.45), and anxiety sensitivity (mean 11.00, SD 5.40). Most participants were highly motivated to quit smoking (Table 1).

**Table 1:** Interview participant characteristics (n=21).

Participant characteristics		Values
Age (years), mean (SD)		47.33 (9.6)
**Age groups (years), n (%)**		
	30-39	6 (29)
	40-49	6 (29)
	50 or more	9 (43)
**Education, n (%)**		
	Less than high-school degree	2 (10)
	High-school graduate or General Educational Development degree	4 (19)
	Some college	6 (29)
	College or graduate degree	9 (43)
**Sex, n** **(%)**		
	Female	14 (67)
	Male	7 (33)
**Ethnicity, n** **(%)**		
	Non-Hispanic	20 (95)
	Hispanic	1 (5)
**Marital status, n** **(%)**		
	Married or living with significant other	3 (14)
	Single or separated	18 (86)
**Employment, n** **(%)**		
	Regular full-time work	7 (33)
	Regular part-time work	3 (14)
	Stay-at-home caregiver	2 (10)
	Unemployed or unable to work	9 (43)
**Average number of cigarettes smoked per day,** **n (%)**		
	10-19	15 (71)
	20 or more	6 (29)
**Years smoked, n** **(%)**		
	10-19	4 (19)
	20-29	12 (57)
	30 or more	5 (24)
**Motivation to quit (range 1-10), n** **(%)**		
	5-7.9	3 (14)
	8-9.9	11 (52)
	10	7 (33)

Before the start of the intervention, participants were asked about their preferred method for quitting smoking. While 17/21 indicated medications (81%) as their preferred cessation method, 11 (52%) also selected smartphone app. Half (n=11) of participants also indicated that the combination of medications and smartphone app would give them the best chance of quitting smoking (Table 2). Following the 6-week intervention, the majority of participants (16/19, 85%) thought that having the app and answering the questions increased their awareness of their smoking behaviors and 20/21 interviewees reported that the app was helpful in quitting smoking. The mean score on the African American Acculturation Scale was 15, indicating a medium level of Black cultural identification [53].

**Table 2:** Interview participant beliefs (n=21).

Participant beliefs				Values
**Preintervention questions, n (%)**				
	**If you were to quit smoking, which of the following would you prefer to receive?**			
		Medications	17 (81)	
		Group counseling	4 (19)	
		Smartphone app	11 (52)	
		In-person individual counseling	6 (29)	
		Smoking cessation telephone helpline	6 (29)	
	**Which option would give you the best chance of quitting smoking?**			
		Medications	4 (19)	
		Both medications and counseling	4 (19)	
		Both smartphone apps and medications	11 (52)	
		Quitting “cold turkey” without counseling or medications	2 (10)	
**Postintervention questions, n (%)**				
	**Consider the number of assessments that were automatically pushed by the smartphone app, was the number of assessments.**			
		Too high	11 (55)	
		About right	8 (40)	
		Not enough	1 (5)	
	**Did carrying the phone and answering questions make you more aware of your thoughts, feelings, and behavior?**			
		Definitely or mostly no	3 (16)	
		Definitely or mostly yes	16 (84)	
	**Overall, how helpful has the smartphone app been in helping you to quit smoking?**			
		Not at all helpful	1 (5)	
		Slightly or moderately helpful	5 (25)	
		Very or extremely helpful	14 (70)	
	**African American Acculturation Scale score range (mean 15, SD 6.5, range 6-27)**			
		Low (score 1-9)	5 (24)	
		Medium (score 10-18)	9 (43)	
		High (score 19-27)	7 (33)	

### Themes

**Figure 2:**
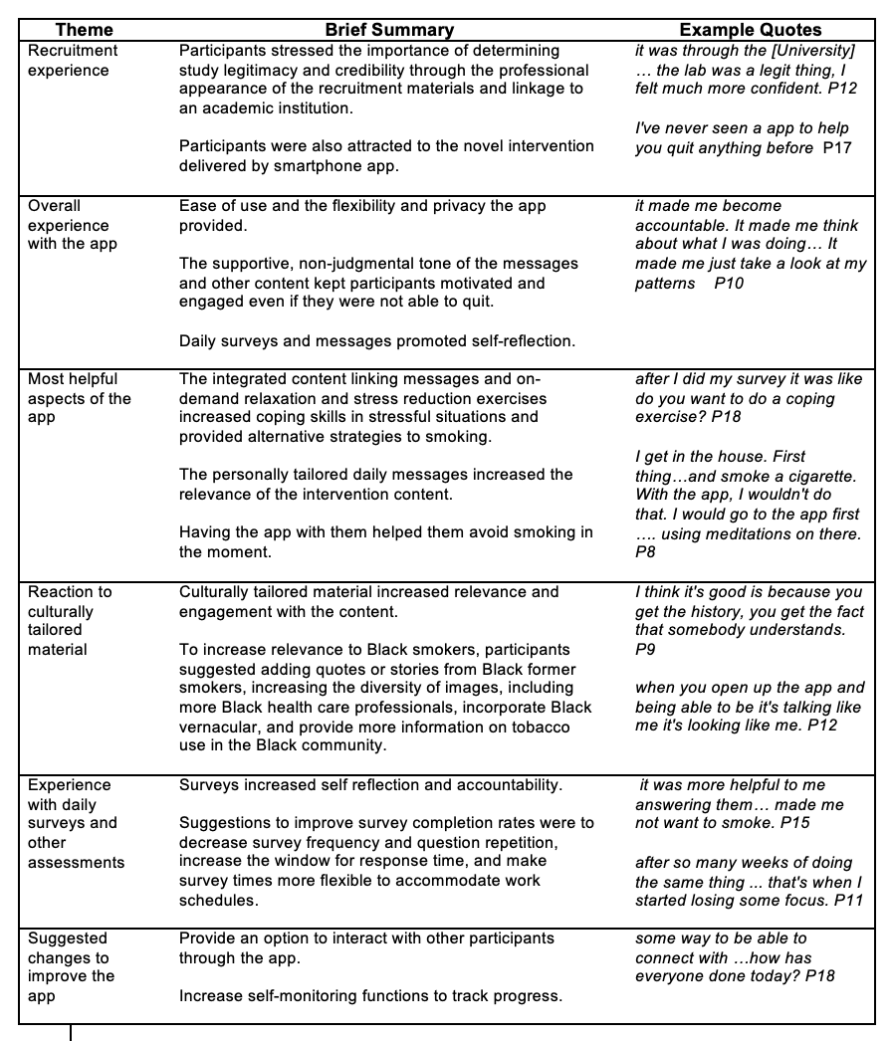
Summary of themes.

Thematic analysis identified 6 themes related to participant experiences using the app which are (1) recruitment experience, (2) overall experience with the app, (3) most helpful aspects of the app, (4) reaction to the culturally-tailored material, (5) experience with the daily surveys and other assessments, and (6) suggested changes to make the app more helpful or relevant to Black people who want to quit smoking (Figure 2 for a summary of themes).

### Recruitment Experience

Participants were asked about their initial reaction to the recruitment materials and what prompted them to complete the screening. Important factors in the recruitment process included the legitimacy of the study, the intervention was delivered using a smartphone app, and the inclusion of more familiar traditional nicotine replacement products.

Participants described the professional appearance of the recruitment materials, direct linkage with a university, and that the screening questions matched the stated purpose of the study as important factors in their initial participation. Participants were positive about being part of a real research study but did not want to be drawn into a for-profit research study. Two participants mentioned that they were skeptical but curious, looking up the university and the specific lab to make sure it was an actual study.

I thought this is probably a scam. But then I thought, okay, well I just won’t give anything too personal, and as I started completing the survey, the questions were, they sounded legit. They were actually about my habit of smoking, and it just sounded scientific enough that I thought, okay yeah, this probably isn’t a scam …. even though it was on Craigslist.

Participant 12 (P12)

Participants were interested in the study when they learned the intervention would be delivered using a smartphone app. This was a new strategy when others had not worked and would allow them more independence during the intervention.

### Overall Experience With the App

Participants commented that they liked the app and found it easy to use and navigate, particularly for adults who are not “tech savvy” or who have not had much experience using smartphone apps. They reported that the app motivated them to keep trying to quit throughout the intervention, even if they were unable to completely quit smoking. Participants also said the supportive, encouraging, nonjudgmental tone of the app was very important to reporting their true behaviors and feelings.

the app … gives you the encouragement of why you don’t have to do that [smoking]. There are other things you can do until you get better at not smoking… to help you along the way during that little minutes when it’s difficult.

P10

it’s not belittling you or anything and still giving you encouragement … they make you change your thought process into a positive one, so I think that’s amazing.

P16

Over half of the participants also said that what they liked most was that the app helped them learn more about themselves and their smoking, made them aware of their smoking behaviors and habits, and “kept me accountable to myself.”

The app made you think about the smoking you were doing. Normally you would just pick up a cigarette and smoke, but the app forces you like OK, do I really need that cigarette right now…. You’re thinking about it more. Well, whereas smoking was just an extension of you before.

P20

About a third of participants mentioned what they liked the most was that the app was something they could do privately rather than with a group of others, trying different options when they were ready and at their own pace.

I was in a group before, and I didn’t really get too much out of it… I’m by myself and I’m doing this alone. I’m with the app, I can think more clearly, and I think this is better than being in the group.

P2

It gives you anonymity. You don’t have to tell anybody…It allowed me to be independent, and not have to speak to someone every day … it just allowed me to do it basically at my pace. I didn’t feel pressured that I had to do it.

P10

A third of the participants mentioned the nicotine replacement products integrated into the intervention.

throughout the day, it would remind me to take more lozenges … that was cool.

P6

### Most Helpful Aspects of the App

Participants found the tailored app content helpful for their current challenges with stress, anxiety, and smoking even if they were not able to quit smoking during the pilot study. Participants commented that the combination of app-directed content and access to complementary on-demand content was a real strength of the intervention. When participants mentioned a specific part of an app that was most helpful to them, they most often mentioned the relaxation and stress reduction exercises and videos, on-demand information that provided them with tips and suggestions, education about tobacco use, and the daily messages (Table 1).

They are targeted to what you put into the app yourself, you would say my anxiety is so much or I’m not able to deal with my anxiety. They would give you tips for that and I thought that was really helpful.

P9

It would tell you after you did the assessments, would you like to take a relaxation or would you like to go or just to go to your toolkit, and that would remind you after you took the assessments to actually do that.

P14

However, if the participant was at work, driving, or busy with family responsibilities, they found it difficult to read or act on a particular message at that time.

Across the different parts of the app, participants said that it was the content related to coping that was particularly helpful, either in the form of a tailored supportive message, developing a new skill to reduce stress or fight cravings, or through exercises to challenge unhelpful thoughts.

I feel like my anxiety leads into anger sometimes and that [the app] makes you sit there and think about it instead of you being in that moment and being so upset that you can’t comprehend what’s going on or be logical about your thinking … Just think about it…let’s do this little activity.

P16

It would pose a question …It would give you a statement and ask you, do you believe this to be true … that really stuck with me. If you smoked one cigarette … do you believe that you could still not smoke the rest of the day … I really believe, once I’ve smoked one, I’m a smoker there’s no stopping… I don’t really challenge my way of thinking…There is a positive side if you would just open your eyes to it.

P12

Others said that the most helpful part of the intervention was having it with them all the time, to use at the moment they needed it.

Remove yourself from the situation… if you can just go to the app in that moment and have that put into your head, that's what’s helpful … because it helps in that moment.

P8

When I was feeling stressed I pushed the button ... just stressed out like where you think you’re gonna light up a cigarette and smoke … you push that button.

P3

### Reactions to Cultural Tailoring

Participants were asked about the cultural tailoring of the app to reflect Black culture. Almost all participants said cultural tailoring contributed to their engagement with the app.

I really like this app being geared toward the Black people, I mean because it is a different thing. The high blood pressure and stress and the discrimination is a totally different thing than what White people face on a daily basis.

P4

Participants were then asked about the relevance of the tailored intervention components to smokers who are Black.

explaining about the history of cigarettes within the African American community, the effect that it had within the community … I feel like it’s very relevant. There’s a lot of facts in there that you wouldn’t even think about as a Black person. There’s a lot of things that were directed towards Black people…they portray it in media. I always thought that it was portrayed more for white people … for a Black person that actually smokes, I think it would be eye opening, it would be very, very helpful for them.

P16

A lot of the tips were directly towards African Americans.

P17

Participant recommendations to increase the relevance of the MASP app for Black people who want to quit smoking included hearing from Black former smokers, either through short videos added to the app, seeing former participant pictures added to the app, or brief quotes or stories from former participants as messages or on-demand content. These pictures and stories could help the participants connect to the project and the app and encourage them during the tough parts of the quitting process.

past users of the actual app that are African American that have been successful with it… if they [participants] have pictures of past people that have actually accomplished this, they'll be more inclined to use it…African American[s] really use this app, his picture’s right there… it worked for him. It might work for me.

P21

Other suggestions to increase relevance for Black people who smoke included more overall diversity in photographs and videos throughout the app, especially more photographs and videos using Black people who smoke and former smokers, as well as Black health care professionals.

It's gotta be by Black people for black people to pay attention... I think a lot of African Americans are, if it's not something that looks familiar or sounds familiar, then they’re scared to go around it.

P12

A few participants suggested additional information on menthol tobacco use that is highly prevalent.

I would just emphasize menthols and maybe Black and Milds? On top of cigarettes, you know that that those are just as deadly.

P18

Several participants also suggested incorporating Black vernacular in videos and messaging.

A more relatable language to the African American community …how we speak like there’s certain words and lingo, maybe just incorporating that… it might appeal to more African Americans... they could read it and be like oh they know what I’m talking about.

P16

### Experience With Daily Surveys and Other Assessments

All of the participants found the surveys easy to complete. Many of the participants thought the daily assessments were a part of the intervention content. Participants described the surveys as holding them more “accountable” and keeping them aware of their smoking behaviors and how much they were thinking about smoking. Several discussed how the survey questions helped them ask for help or remove themselves from a situation where they were more likely to smoke. Most responded quite positively to the idea of the daily surveys, but many participants did not like the amount of repetition of the survey questions and the number of surveys each day. The repetition of the questions over the 6 weeks of the study, which participants acknowledged as necessary to track their progress over time, was often experienced as frustrating or boring in the later weeks of the study.

I knew what questions were coming up and I didn’t even have to read the questions anymore. I was just answering them.

P20

the amount of times you guys asked me a day if I’m depressed …it was a bit excessive. You’re going to get the same answer 2 hours or three hours from now … so you might as well just ask me in the middle of the day … it was exhausting.

P7

Participants provided several suggestions to hold their interest in the surveys and help them complete more of the surveys throughout the study.

a different set of completely different set of questions weekly or change them every day.

P4

have more of a variety of questions…maybe add something, maybe switch them up a little bit.

P18

Participants expressed challenges with what they perceived as a short response window to start the surveys following notification, particularly the surveys that happened during the workday. Participants gave examples of missing surveys because they were driving, with customers, at work, or with family where they could not immediately stop to take the survey or press a button to indicate they would like to take the survey later. Participants also asked for more control over when to take the surveys during the day.

Give us more than five minutes to pull our cars over … there were quite a few times I’m on the highway or I’m on the other line with a doctor’s office or kids school and I need more than five minutes.

P7

### Suggested Changes to Improve the Mobile Anxiety Sensitivity Program for Smoking App

The most commonly suggested improvement was a feature to allow interaction with other participants. Other app improvements included more self-monitoring functions, more pictures and color, and options for longer versions of the training, such as meditation and coping skills.

Hearing from other smokers trying to quit: Participants suggested connecting with other app users to chat, establish a buddy system within the app, or just talk through their challenges and hear from others. They suggested these contacts could take place within the app, be voluntary, and be anonymous to protect participant identities.

set up a buddy system when the person signs up for the study … get permission consent from both parties, if you need to talk to somebody live, this is your buddy.

P10

Maybe like a chat line…go into an open forum. Say we speak to ‘em, just say whatever to chit chat, talk… moral support.

P21

Self-monitoring: Several participants asked if there was a way they could look at their survey data to see their trends in how many cigarettes they were smoking, how they were feeling at the time, and what seemed to trigger their smoking. Other participants suggested an optional journaling function where they could write about their experiences and review their entries later.

The option …to really track your smoking … more on an individual basis so you can actually see …how you were smoking or not or how long you were going without smoking, … especially your progress because I think that's the most motivating to see that you're actually doing it.

P8

## Discussion

### Principal Results

Black people are disproportionally affected by tobacco use and tobacco-related health conditions but historically have been underrepresented in tobacco cessation research [31]. The MASP smoking cessation app was shown to be acceptable and culturally fit with Black adults, and study participants provided recommendations to increase MASP’s relevance to Black people who want to quit smoking.

Given the underrepresentation of Black adults in intervention research, generally, understanding what influences potential participants to complete study screening surveys or to contact study staff members is helpful in increasing participation in clinical trials among Black communities. Participants indicated that it is important that recruitment materials and app content be designed to establish study legitimacy. This includes the professional appearance of recruitment materials and linking the study with a specific university. One recommendation was to include testimonials from previous study participants, which is consistent with other mHealth studies [32,38,41].

Throughout the interviews, participants discussed trust and credibility regarding the intervention and its content. Similar to other studies focused on Black adults, the credibility of the information in the app was often assessed by how well the information in the app matched their own experiences and the source of the information [54,55]. This may be especially important for cultural groups such as Black adults who may experience suspicions or distrust of the medical community due to centuries of historical oppression and systemic racism [56]. To increase credibility and relevance to Black people who smoke, participants recommended including more images reflective of Black adults but also suggested increasing the diversity of the photos and videos beyond Black people to include more photographs and videos of Black health care professionals. However, participants in another study cautioned researchers not to use stock photos or stereotypical images, but to use images and photographs reflective of the app users [32,38].

MASP participants also suggested incorporating more language and statistics on Black people who smoke and more discussion of disparities and targeting by the tobacco industry into the intervention messages to increase engagement and acceptability of the app [32,41]. One novel strategy to increase the number of culturally relevant messages in other mHealth studies involved recruiting a group of Black people who smoke and those who have successfully quit smoking to generate motivational messages for other Black people trying to quit [57-59]. These messages could also be used to incorporate more culturally relevant content and Black vernacular into the app [59]. However, it should be noted that a small number of participants (2 out of 21 in the current study) did not react positively to the explicit cultural tailoring. Diversity of opinions and cultural identity would be expected in any cultural group, so testing messaging and educational content within the app and assessing a participant’s degree of cultural identification at baseline would allow researchers to match culturally relevant messages by level of cultural identification so that participants could receive appropriately tailored messages for their level of cultural identification.

When asked what additional features they would add to the app, participants wanted a way to interact with other participants who were using the app to try to stop smoking. This was interesting, given participants’ comments that they liked using the app because it allowed them privacy and flexibility. Interaction with other study participants within the app may increase cultural relevance as participants would interact with others from their cultural group who were embarking on a shared lived experience of smoking cessation. Other mHealth researchers have suggested a discussion board or group chats moderated by study staff members so that misinformation and unhealthy conversations could not be introduced [27,38]. A discussion board could be an easy option to allow participants to interact with each other [32,41,55,60]. These interactions could also be collected for additional qualitative data analysis. Researchers might want to consider a different discussion board for the control and intervention participants or only for intervention participants. This type of feature has not been well used in previous smoking cessation apps [61,62].

MASP participants also asked for tools that could summarize their progress within the app (eg, cigarette and other tobacco product tracking, money saved, and tracking triggers for smoking). This was also suggested by participants in other mHealth substance use studies [38,41,54,55,63]. Self-tracking or self-monitoring functions could help participants increase their awareness of substance use and personal feelings of accountability for their behavior and develop plans to cope with personal smoking triggers [29]. Both personal accountability and increased awareness of behaviors were named by participants in this and other substance use mHealth studies as something they liked best about the study [54].

MASP participants also recommended prompting fewer surveys per day and adding or substituting questions within the surveys to increase variety and engagement throughout the study. One concern expressed by MASP participants was to make the surveys equally accessible to those who do not have as much control over their daily work schedule, as they cannot always stop what they are doing to complete surveys. This could apply to those who have changing shifts and scheduled work hours, those who drive a lot, are in customer service, and cannot stop serving customers to take surveys immediately, or those who are in hourly positions with less flexibility when they can take short breaks. This was also noted as a challenge in other mHealth interventions [30]. Similarly, MASP participants suggested an option to “try later” for surveys sent when participants could not or did not want to complete them; however, Bendotti et al [55] cautioned app developers to balance additional time to complete surveys with the number of daily notifications from the app as many users did not like frequent notifications. Rather than delaying surveys and messages, another option MASP participants suggested was to allow participants to keep the messages and surveys in a “bank” that they could come back to later that day. However, the MASP app prompted participants with 5 assessments and tailored messages throughout the day, so delaying them until the end of the day would mean participants would not be recording their experiences at the most stressful and potentially most meaningful moments during the day when tailored messages could be most needed to prevent smoking lapse.

### Limitations

Limitations of the study’s qualitative data analysis included the relatively small number of interviews, which limited generalizability to the larger population of Black people who smoke cigarettes. An additional limitation is that the 2 interviewers were not Black. This may have affected participant feedback, both constructive and positive. However, interviewers of the same cultural group as the participants can take unconscious “cultural shortcuts” in communication with participants or make assumptions about participant responses and move too quickly through participant responses without sufficient probing of responses. One suggestion is to have a diverse team of qualitative interviewers and analysts, which may lead to richer data collection and analysis.

### Conclusions

In conclusion, MASP is a feasible and acceptable culturally tailored smoking cessation app for anxiety-sensitive Black adults who want to quit smoking. The development of evidence-based, culturally tailored mHealth apps such as MASP could help to reduce health disparities by improving the cultural relevance of mHealth interventions. Tailored mHealth interventions could decrease barriers to accessing smoking cessation interventions and could increase engagement and acceptability in population subgroups who experience higher burdens of health-harming behaviors and diseases [25,38].

## Appendix

Multimedia Appendix 1 Examples of MASP (Mobile Anxiety Sensitivity Program) app menus.

Multimedia Appendix 2 Sample MASP (Mobile Anxiety Sensitivity Program) video clip.

## Abbreviations

MASP: Mobile Anxiety Sensitivity Program
mHealth: mobile health
NRT: Nicotine Replacement Therapy
