# Supplementary figures and images for: Adapting a Mobile Health App for Smoking Cessation in Black Adults With Anxiety Through an Analysis of the Mobile Anxiety Sensitivity Program Proof-of-Concept Trial: Qualitative Study
Source: JMIR Form Res. 2025 Feb 7;9:e53566. doi: 10.2196/53566 (PMC11845881; doi:10.2196/53566)

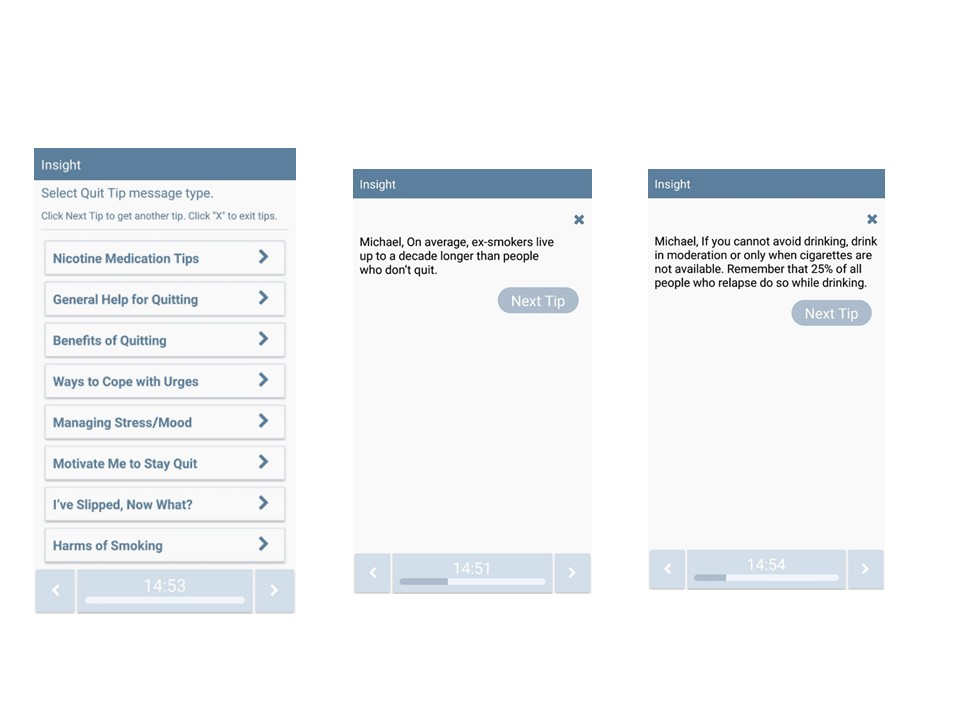

Supplement: Multimedia Appendix 1 [file formative_v9i1e53566_app1.png]
